# Structure-dependent amplification for denoising and background correction in Fourier ptychographic microscopy

**DOI:** 10.1364/OE.403780

**Published:** 2020-11-09

**Authors:** Rémy Claveau, Petru Manescu, Delmiro Fernandez-Reyes, Michael Shaw

**Affiliations:** 1 Department of Computer Science, Faculty of Engineering Sciences, University College London, London WC1E 6BT, United Kingdom; 2 Departement of Paediatrics, College of Medicine of University of Ibadan, Ibadan, Nigeria; 3 Biometrology Group, National Physical Laboratory, Teddington TW11 OLW, United Kingdom; 4 delmiro.fernandez-reyes@ucl.ac.uk; 5 mike.shaw@ucl.ac.uk

## Abstract

Fourier Ptychographic Microscopy (FPM) allows high resolution imaging using iterative phase retrieval to recover an estimate of the complex object from a series of images captured under oblique illumination. FPM is particularly sensitive to noise and uncorrected background signals as it relies on combining information from brightfield and noisy darkfield (DF) images. In this article we consider the impact of different noise sources in FPM and show that inadequate removal of the DF background signal and associated noise are the predominant cause of artefacts in reconstructed images. We propose a simple solution to FPM background correction and denoising that outperforms existing methods in terms of image quality, speed and simplicity, whilst maintaining high spatial resolution and sharpness of the reconstructed image. Our method takes advantage of the data redundancy in real space within the acquired dataset to boost the signal-to-background ratio in the captured DF images, before optimally suppressing background signal. By incorporating differentially denoised images within the classic FPM iterative phase retrieval algorithm, we show that it is possible to achieve efficient removal of background artefacts without suppression of high frequency information. The method is tested using simulated data and experimental images of thin blood films, bone marrow and liver tissue sections. Our approach is non-parametric, requires no prior knowledge of the noise distribution and can be directly applied to other hardware platforms and reconstruction algorithms making it widely applicable in FPM.

## 1. Introduction

Fourier Ptychographic Microscopy (FPM) is a recently developed computational imaging technique that significantly increases the information gathering power of a light microscope. It is based on the fusion of a set of low-resolution (LR) brightfield (BF) and darkfield (DF) images, captured under inclined, spatially coherent illumination. Iterative phase retrieval, whereby the real space amplitude corresponding to the associated coherent passband of an estimate of the extended object spectrum is updated using a recorded image, allows reconstruction of a high-resolution (HR) complex image with an extended field of view [1]. Although originally restricted to the examination of relatively thin samples, recent algorithmic improvements have extended the application of FPM to thicker samples [2–4]. This flexibility, and the low cost of the required hardware [5], make FPM an easy-to-access and readily deployable imaging tool which is particularly attractive for the study and diagnosis of biomedical specimens [6]. However, the performance of all FPM modalities remains fundamentally dependent on the quality of the raw image data; in particular the level of background and associated noise in the DF images. Depending on the numerical aperture (NA) of the objective lens and the required spatial resolution, a typical FPM dataset is comprised of up to 200–300 images of which between 80 and 95% correspond to DF illumination. To minimise image capture time the camera exposure time is usually kept below a few hundred milliseconds which, despite the development of multiplexed illumination strategies [7,8], results in many DF images with a low signal to noise ratio (SNR).

**Fig. 1.**
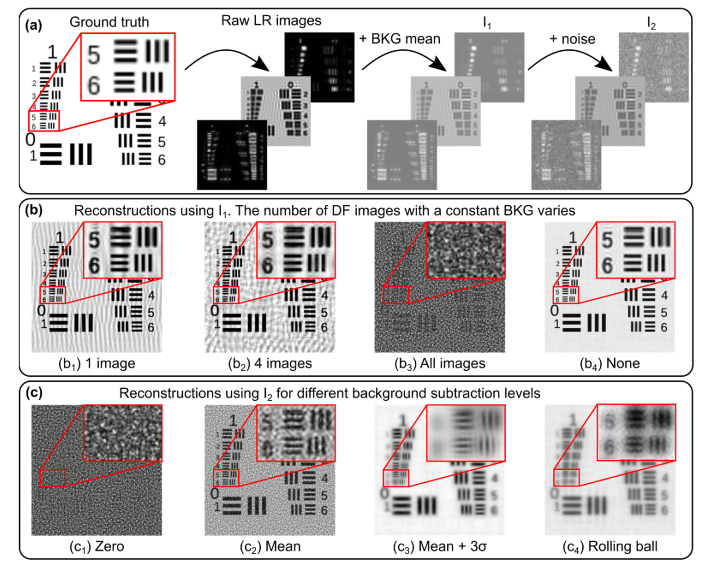
(a) Amplitude of object from which raw LR FPM images were simulated. (b) Amplitude of HR images reconstructed using raw image series I_1_ where a constant non-zero background value has been added to one (b_1_), four (b_2_) and all DF images (b_3_). (b_4_) Amplitude of HR images reconstructed from raw LR (background and noise free) images for comparison. (c) Amplitude of HR images reconstructed from image series I_2_ (degraded with background offset and noise) after subtraction of a background offset of zero (c_1_), the mean pixel value in a structure-free part of the image (c_2_), the mean pixel value plus three times the standard deviation in a structure-free part of the image (c_3_) and with background corrected using the rolling ball method (c_4_).

A critical step in the image reconstruction process is the subtraction of the background in captured DF images. Non-zero background signal in DF images arises from camera dark current and stray light (ambient light and photons multiply scattered within the sample). Adding the information from a DF image with a non-zero background into the estimated HR object spectrum results in spurious signal at a location corresponding to the wavevector of the illumination. In real space this signal appears as bright and dark fringes superimposed on the reconstructed image (Fig. 1(b_1_)). As this is repeated for different dark field images, these fringes sum together coherently to create a high frequency ‘orange peel’ artefact which masks the true structure of the object (Fig. 1(b_2-3_)). In practice, estimation of the background is complicated by the presence of noise, arising primarily from (Gaussian distributed) camera read noise and (Poisson distributed) shot noise. Dark current is also present but negligible for short exposure times, while quantization noise (from the camera’s analogue to digital converter) is typically insignificant compared to read and shot noise [9]. Shot noise increases with the square root of the detected signal (with the SNR similarly dependent) and as a result BF images are generally read noise limited whereas DF images are typically shot noise limited.

Conventionally in FPM DF images are background corrected by subtracting a global background value estimated by averaging the signal within a structure-free region of each DF image [8]. By suitably scaling this background estimate it is possible to effectively remove the noisy background as long as the signal-to-noise ratio (SNR) is large enough. However, as the SNR decreases, fully supressing the background with this approach increasingly results in the removal of signal associated with the structure of the sample. Due to a combination of the angular dependence of the radiant intensity of LEDs typically used in FPM systems [10] and the directional scattering properties of the sample, the SNR decreases with increasing inclination angle. As a result, this simple method tends to be unsuited to removing the background in DF captured with off axis illumination angles, which leads to an inevitable trade-off between image artefacts resulting from insufficient background removal and the loss of the high spatial frequency information associated with these displaced passbands.

To illustrate the effect of inadequate background correction and the presence of noise we simulated a set of 225 FPM LR images from a ground truth HR object corresponding to part of a USAF 1951 resolution test chart shown in Fig. 1(a). The phase of the object was set equal to the object amplitude scaled between 0 and π. A constant offset was added to each simulated LR DF image to account for the average background (image set I_1_). This image set was then further degraded by addition of Poisson and additive white Gaussian noise to approximate real raw image data captured by an FPM system (I_2_). Complex HR images were then reconstructed using the sequential Gauss-Newton algorithm described by Yeh et al. [11]. Figure 1(b) shows images reconstructed from the image set I_1_, with a non zero background offset added to one (b_1_), four (b_2_) or all (b_3_) DF images. The results illustrate an incremental degradation in the HR image as the number of DF images with non-zero background signal increases. In the absence of noise an accurate estimation of background can be easily obtained and subtracting this estimate leads to an artefact-free reconstruction without loss of high frequency details (Fig. 1(b_4_)). However, in the presence of noise this is no longer possible and Fig. 1(c) highlights how simply subtracting a global offset leads to poor quality reconstructed images. If the background threshold is set too low (mean signal within object-free parts of the DF images) the structure in the HR image is obscured by artefacts (c_2_). Setting too high a threshold (mean plus 3 times the standard deviation) removes useful information from the DF images and degrades the spatial resolution of the reconstructed image (c_3_). Assuming the total image noise follows a Gaussian distribution, a reasonable approximation for Poisson noise even at relatively low signal levels [12], this higher threshold level removes 99.7% of the background. In the following we will refer to these background estimates as ‘mean’ and ‘mean + 3σ’. Further simulations showed that picking intermediate background values between these two thresholds slightly improved the reconstructed image quality but invariably resulted in a trade-off between the level of orange peel artefact and the effective spatial resolution. We also used the same simulated FPM image data to investigate the suitability of adaptive methods for removing background in raw DF images, finding that, as with global methods, we were unable to obtain good quality reconstructed images. An example of this is shown in Fig. 1(c_4_) in which the background in each DF image was removed using the rolling ball algorithm [13] prior to reconstruction, where the rolling ball radius was set to a value which gave the best result.

## 2. Previous background subtraction and denoising approaches

To tackle the problems of background correction and noise suppression in FPM, several approaches have been suggested in recent years. Most methods [14–17] are based on modification of the iterative phase retrieval algorithm used in FPM, however this comes at the cost of a significant increase in computational load and complexity. Other methods rely on direct denoising of raw captured images. Fan et al. developed a method [18], in which object signals are separated from noise during the recovery process by iteratively updating a matrix *Cm* defined as the difference between the amplitude of the acquired noisy image and the amplitude of the target image generated from the updated spectrum. Based on the assumption that a pixel of *Cm* whose value is far from 0 is more likely to be noise, the approach consists in setting all the pixel of *Cm* higher than a given threshold to 0 and subsequently modifying the corresponding amplitude image used in the next spectrum update. However, classifying a pixel as object signal or noise is still governed by the subjective choice of a threshold whose value can strongly modify the reconstruction quality. A similar approach was suggested by Hou et al. [19]. It uses an improved thresholding method using a weighting factor, however this leads to balancing the background correction performance and the spatial resolution achieved. Zhang et al. [20] proposed a data pre-processing scheme in which noise reduction is achieved through three successive steps: the removal of stray light in DF images; the correction of uneven background; and the application of a background noise threshold. The choice of this threshold is critical and must be set according to a sensible loss of spatial resolution. Just as with conventional background removal, these approaches lead to an inherent trade-off between spatial resolution and background / noise related artefacts.

**Fig. 2.**
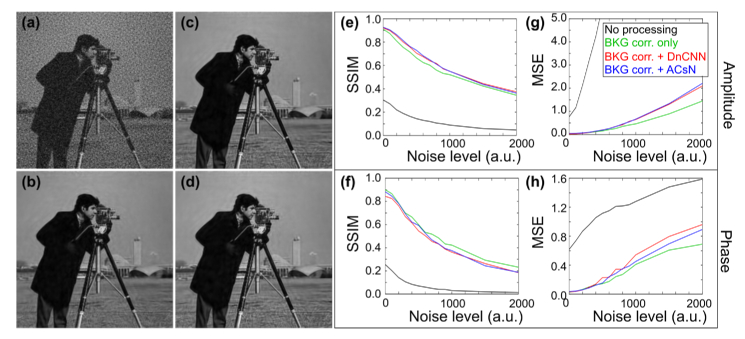
The effect of DF background correction and denoising on reconstructed HR images. (a) Amplitude of reconstructed HR image obtained without denoising, where simulated LR images were degraded with Poisson noise and Gaussian noise with a standard deviation of 500. (b)-(d) Amplitude of reconstructed HR images obtained using different LR pre-processing strategies. (b) The DF background is corrected without further denoising. (c)-(d) LR images are filtered using (c) DnCNN and (d) ACsN algorithms prior to DF background removal to supress noise in parts of the image containing object structures. (e)-(f) SSIM and (g)-(h) MSE metrics for the reconstructed amplitude (top tow) and phase (bottom row) as a function of the Gaussian noise standard deviation. The reference image was obtained from a reconstruction of background and noise-free simulated LR images.

The above methods are based on correction of the DF background through application of a constant threshold across the images, leading to an obvious question: is complete removal of the background, including associated noise, sufficient to reconstruct a high-quality HR image, or is it necessary to suppress noise present in parts of the image corresponding to real object structures? To investigate this, we computationally generated a set of 225 LR images from a complex HR object defined as the standard cameraman test image, where the phase was set equal to the amplitude scaled between 0 and π. We then added an offset to account for background due to stray light and dark current, before adding Poisson noise and different levels of additive white Gaussian noise (Fig. 2). In each case, a background offset was estimated from the mean pixel value in structure-free parts of the DF images and removed. Images were then reconstructed in four different ways. In the first case (Fig. 2(a)) no further background subtraction or denoising was performed. In the second case (Fig. 2 (b)), the residual background signal due to noise was entirely removed by applying a binary object mask to each DF image, where the masks were generated from the noise-free LR DF images in which the background was necessarily zero. In Fig. 2 (c)-(d) DF images were denoised using two state-of-the-art methods, one based on machine learning (Denoising Convolutional Neural Network, DnCNN) [21] and the other an advanced noise correction scheme (Automatic Correction of sCMOS-related Noise, ACsN) [9] developed for fluorescence microscopy, prior to background removal as in Fig. 2(b). Given the similar noise distribution and visual resemblance of fluorescence and DF images, we expect the same denoising method to be applicable in both cases. Each reconstruction (amplitude and phase) was quantitatively compared to the reconstructed ground truth image (obtained without adding any noise) using the structural similarity index measure (SSIM) and the mean square error (MSE).

The results in Fig. 2 clearly demonstrate that artefacts in reconstructed FPM images arise primarily from uncorrected DF background. Figure 2 (e)-(h) show that the reconstructed complex image is of similar quality whether or not denoising of object signals is carried out. We further tested these denoising approaches on real experimental image data and, similarly, found that they did not provide significant additional improvements in image quality. Further, whilst not particularly computationally expensive when processing a single image, denoising an entire set of DF images adds significantly to the overall image reconstruction time. For the data shown in Fig. 2, DnCNN and ACsN denoising added ∼ 5 and 15 seconds to the reconstruction of a 64 × 64 pixel image patch on a standard computer (Intel Xeon CPU E5-1650 processor at 3.20 GHz 16 GB RAM). In contrast, as we will show (section 4), effective DF background correction can be performed in a few ms. Our results suggest that a high quality, artefact-free reconstruction can be simply obtained by effectively removing the DF background without modifying parts of the image corresponding to the underlying object structure. However, as shown in Fig. 1 and also noted by Zhang et al. [20], estimating the DF background is complicated by the presence of noise, resulting in an inherent trade-off between artefact minimisation and attenuation of real DF image information.

## 3. Description of the SdA algorithm

**Fig. 3.**
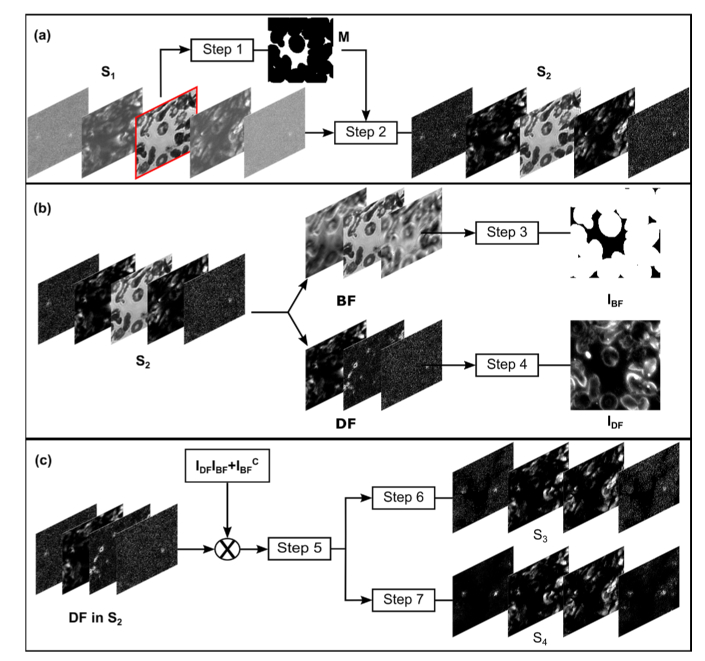
Flow-chart illustrating the principal steps in the SdA correction method. (a) A binary image mask (M), created by OTSU binarization of the on-axis BF image, is used to perform initial background subtraction of DF images and leading to a new set of images (S_2_). (b) BF and DF images in S_2_ are used to generate two intermediate images, I_BF_ and I_DF_. (c) I_BF_ and I_DF_ are combined in a scaling term used to amplify object pixels in DF images within S_2_. After further background correction (step 5) the images are rescaled to create two further image sets, S_3_ and S_4_. A final reconstructed FPM image is computed by combining images in S_3_ and S_4_ within the FPM iterative phase retrieval algorithm.

To solve the problem of effective background removal in FPM we developed a new approach based on Structure-dependent Amplification (SdA). The method consists of classifying image pixels as belonging to ‘object’ or ‘background’ classes according to whether or not they correspond to regions of the underlying object containing structures (i.e. scatterers which give rise to contrast in DF images). The pixel values within these two classes are modified differentially in the raw FPM images, which has the effect of relaxing the critical estimation of a background threshold. In this way the background (and associated noise) is removed without attenuating the information contained in the DF images. The method comprises three steps as shown diagrammatically in Fig. 3. First (Fig. 3(a)), the entire dataset (S_1_) is processed to remove the mean background pixel value, which is estimated from structure-free areas in the DF images. Next (Fig. 3(b)), we take advantage of the data redundancy in real space in both the BF and DF subsets to create two intermediate images, *I_BF_* and *I_DF_*, which serve to map and amplify the useful information contained in the DF images. Finally (Fig. 3(c)) *I_BF_* and *I_DF_* are used to modify the intensity of the DF images, leaving the background pixels at their original value so that they can be easily filtered out. In this final step the filtering of noisy pixels is performed in two slightly different ways producing two new sets of differentially denoised DF images (S_3_ and S_4_ in Fig. 3) which are both incorporated into the final reconstructed image through the iterative phase retrieval algorithm. Overall, the method classifies pixels as ‘background’ or ‘object’, boosts the value of ‘object’ pixels, suppresses the influence of pixels identified as ‘background’ and finally rescales ‘object’ pixels to their original value.

### 3.1. Initial background correction (a)

*Step 1*: Image pixels are coarsely classified as ‘object’ or ‘background’ based on Otsu binarization of the normal incidence BF image, *I_0,0_*. A mask *M* is then generated by morphological dilatation (⊕) of the ‘0’ regions in the resulting binary image using a disc shaped structuring element *s_1_* to reclassify object pixels originally included in the background:

(1) $$M=s_{1}⊕thresh(I_{0,0})\;.$$

Within reasonable limits (10–40 pixels) the quality of the reconstructed image is not especially sensitive to the size of the structuring element, however we found a radius of 20 pixels gave optimal results.

*Step 2*: A new set of images, S_2_, is created by subtracting the mean background in each DF image in S_1_ using the binary mask *M* calculated in step 1. Negative values are set to 0.

(2) $$S_{2}=\max\{S_{1}-mean[S_{1}(M)]\;\;,\;\;0\}\;.$$

### 3.2. Mapping of the ‘object’ and ‘background’ regions (b)

*Step 3*: The image *I_BF_* is obtained by taking the reciprocal of the sum of all BF images. Residual noise is entirely suppressed by subtracting the maximal value within the background area *M* and the positive and negative pixels are respectively set to 1 and 0. Fine morphological dilatation using a disc shaped structuring element s_2_ (∼ 8 pixels radius) is applied to fill holes and weak edges missed in the binarization operation (Eq. (3)). Whilst *I_BF_* provides a more accurate classification of image pixels into object and background classes than *M*, the morphological dilatation, necessary to prevent misclassification of object pixels, means that background pixels around the edges of foreground objects remain

(3) $$\begin{array}{l} I_{BF}={\left(\sum_{i\in BF}S_{2}^{i}\right)}^{-1}-\max\left[{\left(\sum_{i\in BF}S_{2}^{i}\right)}^{-1}\cdot M\right]\\ I_{BF}(x,y)=\left\{ \begin{array}{l} 1\;\;\;\;\;\;\text{for}\;I_{BF}(x,y)>0\\ 0\;\;\;\;\;\text{otherwise} \end{array}\right.\\ I_{BF}=s_{2}⊕I_{BF}\;. \end{array}$$

*Step 4*: A further intermediate image *I_DF_* is computed as the sum over all DF images minus the mean pixel value in *I_DF_*× *M*:

(4) $$I_{DF}=\max\left\{\sum_{i\in DF}S_{2}^{i}-mean\left[\sum_{i\in DF}S_{2}^{i}(M)\right]\;\;,\;\;0\right\}\;.$$

### 3.3. Amplification and final background correction (c)

*Step 5*: After amplification of the ‘object’ pixels by multiplication by *(I_DF_I_BF_ + I_BF_^C^)*, where *I_BF_^C^* is the complement of *I_BF_* (Eq. (5)), DF images are further background corrected by subtraction of the mean value of the background pixels plus three times the standard deviation. Including the *I_BF_^C^* term in the multiplication factor ensures that this operation only scales the image intensity without removing the background and potentially low value object pixels. This then allows removal of the background whilst minimising the suppression of object information:

(5) $$S_{2}=S_{2}\cdot (I_{DF}I_{BF}+I_{BF}^{C}),$$

(6) $$S_{2}^{\prime }=\max\{S_{2}\cdot I_{BF}-(mean[S_{2}(M)]\;\;+3\times std[S_{2}(M)])\;\;,\;\;0\}\;.$$

*Step 6*: S_3_ is obtained by reassigning the non-zero pixels to their original intensity values, meaning that any positive pixels will still be present in the image with the same intensity:

(7) $$S_{3}=\left\{ \begin{array}{l} S_{2},\;\;\;\;\text{if }{S^{\prime }}_{2}>0\\ 0,\text{ otherwise} \end{array}\right.\;\;.$$

**Fig. 4.**
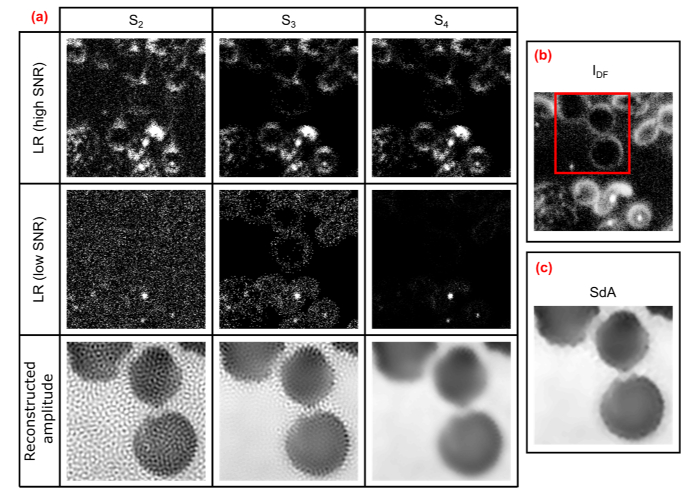
Intermediate and final reconstructed images generated using the SdA method. (a) Comparison of LR DF images in the series S_2_, S_3_ and S_4_ and the corresponding HR reconstructed images. (b) Image I_DF_ used to amplify ‘object’ pixels in S_3_ and S_4_. (c) Reconstructed image using SdA, which incorporates a combination of S_3_ and S_4_ in the iterative phase retrieval algorithm. The HR images correspond to the red square highlighted in (b).

This direct reassignment retains all the information in the ‘object’ pixels, however it can be problematic for DF images with a very low signal to background ratio (SBR, defined as the ratio of the mean pixel value for object pixels to background pixels). For such images, the signal contained within the object pixels is very small and often one (even several) order of magnitude smaller than the background noise. Commonly only a few object pixels have sufficient intensity to be distinguishable from the background. As a result, the multiplication by *(I_DF_I_BF_ + I_BF_^C^)* often leads to amplifying regions in the image where the ‘object’ information is completely buried within the background noise, resulting in a reconstructed image that includes strong artefacts around the edges of foreground objects (second column in Fig. 4(a)).

*Step 7*: To correct this problem, a second series S_4_ of processed images is generated. Instead of restoring the original intensity for all non-zero pixels, each DF image with a low (SBR), less than 1, is normalised with respect to its maximal value (intensity of an object pixel) and then multiplied by the maximal value of the same image in S_2_:

(8) $$S_{4}=\left\{ \begin{array}{ll} S_{2}, & \text{ if }{S^{\prime }}_{2}>0\text{ and }SBR>1\\ \frac{{S^{\prime }}_{2}}{\max({S^{\prime }}_{2})}\cdot \max(S_{2}) & \text{ if }{S^{\prime }}_{2}>0\text{ and }SBR<1\\ 0, & \text{ otherwise} \end{array}\right.\;\;.$$

In this way the influence of residual background pixels is strongly reduced, however the resulting reconstructed image suffers from decreased spatial resolution (third column in Fig. 4(a)).

To yield an optimal reconstruction, SdA uses both S_3_ and S_4_, incorporating images from S_4_ for the first N-1 iterations of the phase retrieval algorithm and S_3_ for the final iteration. This enables an efficient removal of pattern artefacts introduced by the DF background while retaining high frequency information contained within DF images (Fig. 4(c)).

### 3.4. Further post-processing

For very occasional cases where the raw images are extremely noisy, slight residual artefacts may still be observed around object edges. These can be very easily corrected using a local median filter directly applied on the reconstructed image. Specifically, we use *I_BF_* and the binarization of the reconstructed image amplitude to precisely locate the area to filter.

(9) $$\begin{array}{l} R=I_{BF}-s_{3}⊕thresh{\left(I\right)}^{C}\\ I_{corr}=I\times R^{C}+medianFilter(I)\times R\;, \end{array}$$

with *R* the region to filter, *s_3_* a 3 pixels radius structuring element and *I* the reconstructed image amplitude. The dilatation operation makes sure that the filtering does not introduce unnecessary blurring of object edges.

## 4. Results

### 4.1. Simulations

**Fig. 5.**
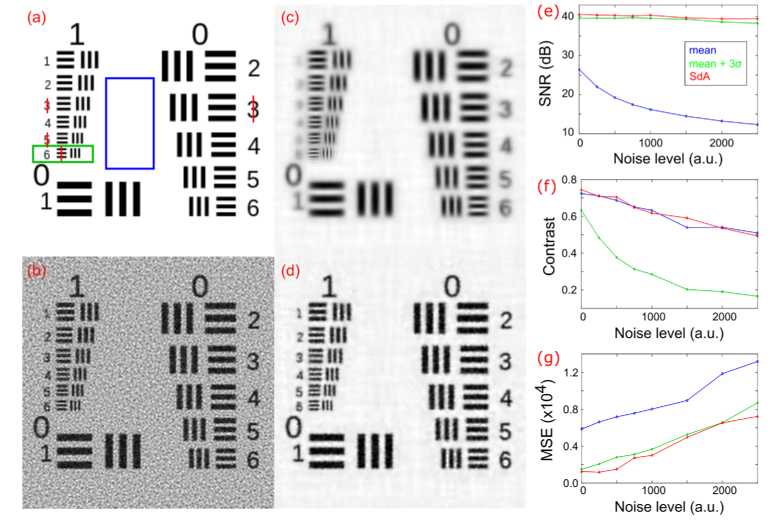
Comparison of reconstructed HR images obtained using different background correction methods. (a) Amplitude of ground truth HR object. (b)-(d) Reconstructed HR amplitude images obtained by pre-processing the LR images by subtraction of ‘mean’ (b) ‘mean + 3σ’ (c) background estimates and the SdA method (d). For (b)-(d) the simulated LR images were degraded with Poisson noise and additive Gaussian noise with a standard deviation of 1000. (e)-(g) Quality metrics for images reconstructed using the three methods for different levels of Gaussian noise. (e) SNR assessed within the blue rectangle in (a). (f) Average contrast measured along the red lines in (a). (g) MSE in the green rectangle cropped region of each reconstruction compared to the ground truth in (a).

To test the performance of the SdA method we computed a set of LR FPM images from a complex object based on an HR image of part of a USAF resolution test target (Fig. 5(a)), where the phase of the object was set to the amplitude scaled between 0 and π. As previously, after adding an offset to account for mean background signal caused by stray light and dark current, Poisson noise and different amounts of additive white Gaussian noise were added to simulate real raw data. To assess the performance of our method, we quantitatively compared the HR reconstructed images to those obtained using the ‘mean’ and ‘mean + 3σ’ background correction approaches.

Visually, we observe from Fig. 5(a-d) that SdA leads to a reconstruction of better quality compared to the conventional approaches. The mean background subtraction method fails to remove the DF background resulting in the characteristic speckle structure in Fig. 5(b). Subtracting a mean + 3σ background value from the raw DF images removes real image information reducing effective spatial resolution which leads to a blurry image in Fig. 5(c). These observations are consistent with the SNR and contrast measurements shown in Figs. 5(e) and (f). The SNR was assessed in a homogeneous region of the reconstructed image (blue rectangle in Fig. 5 (a)) and defined as the logarithmic ratio of the average pixel value to the standard deviation. We observe a similar and almost constant SNR for the ‘mean + 3σ’ and SdA approaches even in the presence of significant noise in LR raw images. On the other hand, because the background denoising threshold used in the ‘mean’ approach is too low, the reconstructions become severely degraded as the noise level in the LR raw images increases. Figure 5(f) shows that the image contrast, measured across the red line profiles in Fig. 5(a), is significantly lower for the ‘mean + 3σ’ background subtraction method, whilst images reconstructed using the ‘mean’ and SdA methods maintain high contrast because the high spatial frequency information contained in the raw DF images is not removed during the background subtraction process. By retaining high frequency information contained within noisy DF images, whilst still effectively removing background signal, SdA leads to high quality, high resolution reconstructed images. Figure 5(g) shows the MSE between each reconstruction and the reference HR object within the green cropping window shown in Fig. 5(a). Again, by effectively removing the noisy background without attenuating real signal SdA outperforms conventional global background subtraction at all level of noise.

### 4.2. Experimental results

**Fig. 6.**
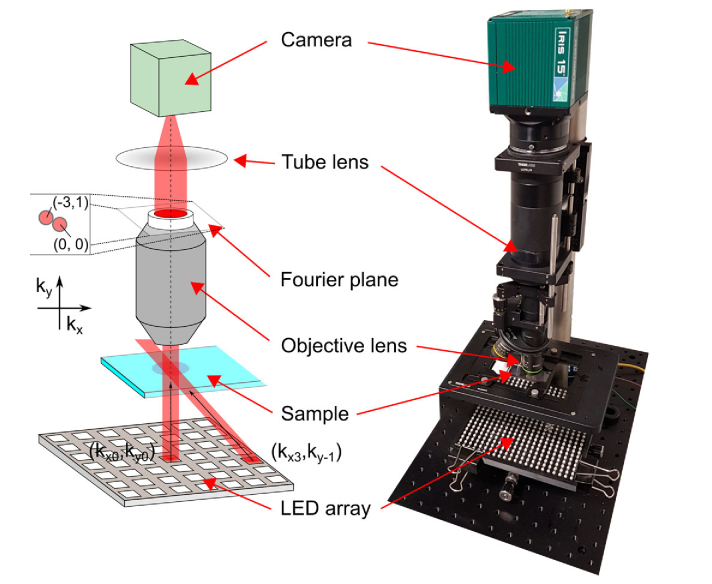
Experimental FPM system used to generate image data to test background correction methods. (left) schematic diagrams showing illumination from two different LEDs to illustrate the displacement of the passband centre in the Fourier (pupil) plane of the objective lens. (Right) photograph of the microscope.

To further test the performance of the SdA method, raw image sets were captured using an in-house built FPM system composed of an array of 22 × 22 addressable RGB LEDs (BTF-lighting), an air immersion objective lens (4x/0.16, 10x/0.3 or 20x/0.45 – UPLSAPO4x, MPLFLN10x, MPLFLN20x Olympus), a tube lens with a focal length of 200 mm (TTL200-A, Thorlabs Inc.) and a digital camera (Iris 15, Photometrics) with 5056 × 2960 4.25 µm pixels [22] (Fig. 6). All images were acquired using µ-manager software with the switching of LEDs synchronized to the global exposure period of the camera’s rolling shutter using a microcontroller (Arduino Uno). Each image set contained 225 images captured under sequential illumination of the sample with the LEDs arranged inside a filled circle of diameter 11.2 cm. With an exposure time of 100 ms, the total acquisition time was slightly less than 30 seconds per image set.

After reconstruction an extended depth of field image was computed to optimally display 3D information within a single 2D image, by numerical propagation of the recovered complex field [22]. Due to wavefront curvature, a subset of raw images contained both BF and DF regions within the same image. These images are particularly problematic from a background correction / denoising perspective and, as a result, were automatically detected and excluded from the image set prior to reconstruction. With a sufficient spectral overlap between adjacent captured images (as in our case) removing these images does not result in gaps in the recovered object spectrum. Where the overlap between adjacent passband is not sufficient these hybrid BF/DF images can be treated using approach described by Zhang et al. [20], in which the BF area in the captured image is replaced by the corresponding DF area of the target image obtained from the updated spectrum.

**Fig. 7.**
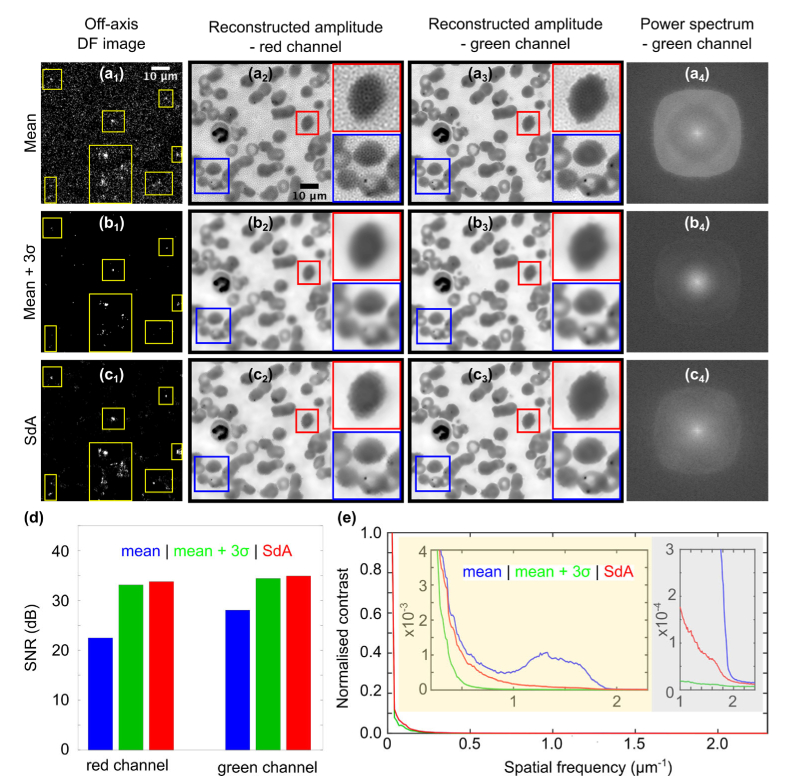
Experimental comparison between results obtained using ‘mean’ (a), ‘mean + 3σ’ (b) and SdA (image series S_4_) (c) background correction approaches. (a_1_)-(c­_1_) DF images corresponding to the largest off axis illumination angle. Yellow boxes indicate the locations of pixels containing useful signals. HR reconstructions of the red (a_2_)-(c_2_) and green (a_3_)-(c­_3_) channels. (a_4_)-(c­_4_) Power spectra of reconstructed green channel amplitude images. (d) SNR of each reconstructed image. (e) Azimuthally averaged power spectrum of (a_4_)-(c­_4_) illustrating the extent of the frequency support for images reconstructed using the different methods.

We tested the performance of SdA background correction on Giemsa stained peripheral blood thin film samples taken from a patient infected with *P. falciparum* malaria parasites. Images were captured using a 10x/0.3 objective lens at two different illumination wavelengths, 530 nm and 632 nm. Figure 7 shows the results obtained using ‘mean’ (first row), ‘mean + 3σ’ (second row) and SdA (third row) background correction methods. Figure 7(a_1_)-(c_1_) shows raw DF images at 632 nm captured at the maximum off axis illumination angle demonstrating how only SdA (a_3_) is able to retain object information (yellow boxes) while effectively suppressing the background in noisy images. Figure 7(a_2_)-(c_2_) and (a_3_)-(c_3_) show the reconstructions of the red and green channels respectively, illustrating the differences in reconstruction quality for different level of noise. The SNR is significantly higher in the green channel due to a combination of the higher brightness of the green LEDs and the higher quantum efficiency of the camera at 530 nm. Visually, SdA reconstructed images are free from the background associated artefacts apparent in images reconstructed using ‘mean’ background subtraction. By retaining high spatial frequency information, image sharpness is much improved compared to the ‘mean + 3σ’ method. The figure insets (red boxes in (a_2_)-(c_3_)) show how reconstruction artefacts prevent clear visualization of the (spiculated) cell morphology. The fine projections from the cell are clearly visible in images reconstructed using SdA (Fig. 7(c_3_)). Furthermore, the malaria parasites (dark dots in the blue insets) are barely visible in images reconstructed using the ‘mean + 3σ’ method, but are clearly visible in SdA reconstructed images. Figure 7(a_4_)-(c_4_) show the corresponding logarithmic power spectra of Fig. 7(a_3_)-(c_3_). For the ‘mean’ method (a_4_) a lot of energy is concentrated around the edges of the nominal passband due to high-frequency noise artefacts in the reconstructed image. This signal is not present for the ‘mean + 3σ’ method due to the aggressive background removal, however almost all of the energy is concentrated at low frequencies due to loss of high frequency information from the DF images. By contrast, images reconstructed using SdA (Fig. 7(c_4_)) display a gradual monotonic reduction in spectral power with increasing frequency as expected for a natural HR image. To quantitatively assess the reconstruction quality, we estimated the SNR and the spatial resolution for the three methods. The SNR (Fig. 7(d)) was measured for the red and green channels in the background area, with results indicating an improvement of ∼ 11dB and 7dB respectively using SdA compared to the ‘mean’ method. Figure 7(f) shows the azimuthally averaged power spectra shown in Fig. 7(a_4_)-(c_4_). SdA maintains the spatial frequency cut-off at 2 µm^-1^ and increases the contrast at intermediate to high spatial frequencies compared to ‘mean + 3σ’ background subtraction, whilst removing the spurious high frequency signals due to noise in images reconstructed using ‘mean’ background subtraction (bump from 1 to 1.75 µm^-1^ in the blue curve). In terms of additional processing time, running the SdA algorithm on a standard lab workstation added an average of 70 ms to the total reconstruction time for a series of 225 images, 200 × 200 pixels in size.

**Fig. 8.**
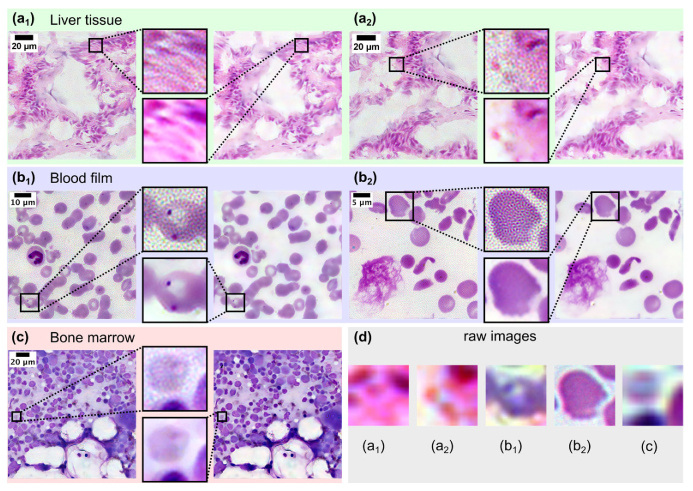
Comparison of colour reconstructions using SdA (right hand-side) and the global ‘mean’ (left hand-side) background removal. (a_1-2_) Liver tissue section. (b_1-2_) Thin blood film. (c) Bone marrow section. (a_1-2_) and (c) were captured using a 4x/0.16 objective lens with a reconstructed (synthetic) NA of 0.7. (b_1_) and (b_2_) were captured using 10x/0.3 and 20x/0.45 objective lenses with synthetic NAs of 0.9 and 1.15 respectively. (d) Raw brightfield images captured at normal incidence corresponding to each close-up view for comparison.

Finally, we also applied SdA to reconstruct colour FPM images of different biomedical samples acquired by sequential capture of raw image sets under illumination at 475 nm, 530 nm and 632 nm (Fig. 8). Lateral and axial chromatic offsets were corrected using lateral image registration and post reconstruction refocusing [22] before the three colour channels were merged and white balanced to yield an RGB colour image. Images were captured using different objective lenses (depending on required field of view and spatial resolution). A 4x/0.16 lens was used to acquire images of 5 µm thick haematoxylin and eosin stained liver tissue and ∼7 µm thick May-Grűnwald-Giemsa stained bone marrow sections and 10x/0.3 and 20x/0.45 lenses were used to image Giemsa stained peripheral blood films.

In all cases applying SdA significantly improves the quality of the reconstructed image. Close-up views in Fig. 8 clearly show the characteristic high frequency speckle / orange peel pattern superimposed on the image (top insets), which is completely absent when SdA is applied (bottom insets). This enables a clearer visualisation of tissue morphology (Fig. 8(a_1_)). It also improves visualisation of cellular morphology, as observed in Fig. 8(b_2_), where the spiculated projections from the cell membrane are more clearly visible after denoising. Whilst less striking, the edges of the cell highlighted in Fig. 8(c) are more clearly defined facilitating further analysis such as segmentation. Close inspection of the denoised image in Fig. 8(a_2_) also reveals several distinct spot-like structures which are masked by high frequency speckle in the noisy HR image. Similarly in Fig. 8(b_1_) the cytoplasmic ring of the malarial parasite in the upper part of the cell is only visible in the SdA reconstructed image. Such fine morphological features are of critical importance for diagnostic applications [23].

## 5. Conclusion

We have developed a conceptually simple method that efficiently performs background subtraction in raw FPM datasets and substantially improves the quality of reconstructed high-resolution images. By removing the need for selection of a background noise threshold, which is required in many conventional background correction and denoising approaches, our (SdA) method obviates the traditional spatial resolution / artefact suppression trade-off in FPM that results from the low SNR of DF images captured at large illumination angles. We have shown through both simulated and experimental data that SdA removes background and noise-related image artefacts and increases the SNR of reconstructed images without the sacrifice of high frequency information, which results from overcorrection of the DF background; essential for the reconstruction of fine sample details such as cell morphology and the visualisation of small objects (such as malarial parasites). Implementation of SdA relies on a set of simple image processing operations and does not involve any modification to the classic FPM phase retrieval algorithm. As a result, the computational effort required is negligible and the method adds only a few tens of milliseconds to the overall image reconstruction time (which is typically around one minute). SdA relies only on the data redundancy in the raw image set and requires no assumptions about the background level or noise distribution, making it easily adaptable to any FPM platform. While demonstrated for sequential capture, SdA can just as easily be applied to FPM data acquired using a spectral or spatial multiplexing illumination strategy. Most artefacts in reconstructed FPM images arise from under or over estimation of the DF background (and associated noise). As a result we found that further denoising of object pixels yielded no significant improvement in reconstructed image quality and at far greater computational expense and execution time. By rapidly and effectively removing artefacts whilst retaining real image information SdA enables fast, high resolution, artefact free FPM imaging for a wide range of applications.
